# Genome-wide identification, characterization, and evolutionary analysis of the *HSP70* gene family in rice (*Oryza sativa* L.)

**DOI:** 10.1186/s12870-025-07574-8

**Published:** 2025-12-01

**Authors:** Nagy S. Radwan, Sobhi F. Lamlom, Abdul-Hamid Emwas, Mariusz Jaremko, Nader R. Abdelsalam

**Affiliations:** 1 Agricultural Botany Department, Faculty of Agriculture, Saba Basha, Alexandria University, Alexandria, 21531 Egypt; 2 Department of Plant Production, Faculty of Agriculture Saba Basha, Alexandria University, 21531 Alexandria, Egypt; 3 Core Lab of NMR, King Abdullah University of Science and Technology (KAUST), Thuwal, Makkah, 23955-6900 Saudi Arabia; 4 Division of Biological and Environmental Sciences and Engineering (BESE), Smart-Health Initiative (SHI) and Red Sea Research Center (RSRC), King Abdullah University of Science and Technology (KAUST), Thuwal, Makkah, 23955-6900 Saudi Arabia

**Keywords:** Heat shock protein 70, Stress tolerance, Gene expression, Evolutionary genomics, Climate resilience

## Abstract

Heat shock proteins 70 (*HSP70*s) are highly conserved molecular chaperones essential for maintaining protein homeostasis under environmental stress conditions. Despite rice being a staple crop for over half the world population and facing increasing climate-related stresses, comprehensive characterization of its *OsHSP70* gene family has remained incomplete. We identified and characterized 32 *OsHSP70* genes in the rice genome through integrated bioinformatics and experimental approaches. Phylogenetic analysis classified these genes into five distinct subfamilies (A-E), with subfamily D predominating (15 members, 46.9%). Chromosome mapping revealed non-random distribution across 9 chromosomes, with notable gene clusters on chromosomes 3 (7 genes) and 11 (10 genes), indicating tandem duplication as a major expansion mechanism. Evolutionary analysis through Ka/Ks ratios demonstrated that 83% of duplicated gene pairs evolved under purifying selection (Ka/Ks < 1), with divergence times spanning from 0.98 to 64.75 million years ago. Subcellular localization predictions identified diverse targeting patterns: 34.9% chloroplast, 32.6% cytoplasm, and 14.0% mitochondria, reflecting functional compartmentalization. Quantitative RT-PCR analysis under five abiotic stress conditions (heat, cold, drought, salt, and submergence) identified seven highly stress-responsive genes (*Os01g62290*, *Os03g02260*, *Os03g11910*, *Os03g16860*, *Os03g16920*, *Os03g50250*, *Os05g35400*, and *Os05g38530*) with maximum fold changes exceeding eightfold, particularly under heat stress. *Os03g50250* (*OsHSP70-13*) emerged as the strongest responder across multiple stresses. Protein–protein interaction networks revealed integration with key metabolic and stress response pathways. These findings establish a comprehensive framework for understanding *OsHSP70*-mediated stress tolerance mechanisms in rice and identify priority candidates for developing climate-resilient varieties through targeted breeding or genome editing approaches.

**Supplementary Information:**

The online version contains supplementary material available at 10.1186/s12870-025-07574-8.

## Introduction

Climate change poses unprecedented challenges to global agriculture, with rising temperatures, increased drought frequency, and extreme weather events threatening food security worldwide. Abiotic stresses account for approximately 70% of annual crop yield losses, with temperature extremes alone reducing rice productivity by 10% per decade [1]. As the primary staple food for over half the world population, rice (*Oryza sativa* L.) productivity is critical for feeding an estimated 9.7 billion people by 2050 [2, 3]. Understanding the molecular mechanisms underlying stress tolerance in rice is therefore essential for developing climate-resilient varieties capable of maintaining yields under increasingly challenging environmental conditions [4].

Plants have evolved sophisticated molecular defense systems to cope with environmental stresses, among which heat shock proteins (HSPs) represent one of the most ancient and conserved cellular protection mechanisms [5–7]. HSPs function as molecular chaperones that maintain protein homeostasis under stress conditions by preventing protein aggregation, facilitating proper protein folding, and assisting in the degradation of irreversibly damaged proteins [8]. Among the HSP families, *HSP70* proteins are particularly crucial, serving as primary gatekeepers of cellular protein quality control through their ATP-dependent chaperone activities [9]. These proteins operate through a sophisticated mechanism involving an N-terminal nucleotide-binding domain (NBD) and a C-terminal substrate-binding domain (SBD), enabling them to recognize misfolded proteins and facilitate their proper refolding or degradation [10–13].

The evolutionary significance of *HSP70* proteins in plant adaptation becomes evident when examining their expansion patterns across plant genomes [3]. Unlike their animal and microbial counterparts, plant *HSP70* families have undergone extensive diversification through whole-genome and segmental duplications, resulting in specialized subfamilies with distinct subcellular localizations and stress-responsive functions [14–16]. This diversification has enabled plants to develop sophisticated stress response networks, with HSP70 proteins functioning not only as molecular chaperones but also as key regulators of stress signaling pathways [3, 16–18]. Plant HSP70s are typically divided into several subfamilies based on their subcellular localization, including cytosolic, endoplasmic reticulum (ER), mitochondrial, and chloroplastic forms, each playing specialized roles in maintaining proteostasis within their respective compartments [16].

Recent advances in plant molecular biology have revealed that *HSP70* proteins play multifaceted roles extending beyond protein folding. They participate in hormone signaling cascades, particularly abscisic acid (ABA) responses during drought stress [16, 19, 20]. Moreover, *HSP70* proteins exhibit complex developmental regulation, with specific family members showing tissue-specific expression patterns during reproductive development [21–23]. Emerging evidence also suggests their involvement in plant-pathogen interactions and RNA virus multiplication, indicating broader roles in plant immunity [24]. This functional diversity underscores the importance of comprehensive characterization of *HSP70* gene families in economically important crops.

Genome-wide analyses of *HSP70* gene families have been conducted in various plant species, revealing considerable variation in gene family size and complexity. Previous studies have identified 18 *HSP70* genes in *Arabidopsis thaliana* [25], 21 in pepper (*Capsicum annuum*) [26], 20 in potato (*Solanum tuberosum*) [27], 21 in pumpkin (*Cucurbita moschata*) [13], 34 in radish (*Raphanus sativus*) [28], 52 in cabbage (*Brassica oleracea*) [29], 61 in tobacco (*Nicotiana tabacum*) [30], and 24 in common bean (*Phaseolus vulgaris*) [31]. These variations reflect differences in genome sizes, polyploidy events, and evolutionary histories among different plant lineages. In rice, although the *HSP70* gene family was previously identified [32], detailed functional characterization, particularly regarding expression responses to multiple abiotic stresses, remains limited.

Rice is highly vulnerable to various abiotic stresses, including drought, salinity, extreme temperatures, and heavy metal pollution, all of which are expected to increase in frequency, intensity, and duration due to climate change [32–34]. This vulnerability threatens rice productivity and consequently global food security. Despite the recognized importance of HSP70 proteins in stress tolerance, comprehensive integrative analyses combining genomic characterization, evolutionary dynamics, and experimental validation of stress-responsive expression patterns in rice have been lacking. Such information is crucial for identifying candidate genes that could be targeted for crop improvement through conventional breeding, marker-assisted selection, or genome editing technologies.

To address these knowledge gaps, we conducted a comprehensive genome-wide analysis of the *HSP70* gene family in rice, integrating bioinformatics approaches with experimental validation. Our objectives were to identify and characterize all *OsHSP70* family members in the rice genome; analyze their phylogenetic relationships, gene structures, and evolutionary dynamics; predict subcellular localizations and protein–protein interaction networks; experimentally validate expression patterns under multiple abiotic stress conditions including heat, cold, drought, salt, and submergence; and identify priority candidate genes for future functional studies and crop improvement efforts. This integrated approach provides fundamental insights into *OsHSP70* gene family organization and establishes a framework for developing climate-resilient rice varieties through targeted molecular breeding and biotechnological strategies.

## Materials and methods

### Genome data retrieval and *OsHSP70* gene identification

The complete genome sequence and annotation files (GFF3 format) of *Oryza sativa* (Nipponbare, MSU Rice Genome Annotation Project Release 7.0) were downloaded from the Phytozome database v13.0 (https://phytozome-next.jgi.doe.gov/). To identify *OsHSP70* family genes, we employed a dual approach that combined Hidden Markov Model (HMM) profiling and BLAST searches. The *OsHSP70* domain profile (PF00012) was obtained from the Pfam database v35.0 (http://pfam.xfam.org/) and employed for HMM-based searches using HMMER v3.3.2 implemented in TBtools v1.120 [29]. HMM searches were conducted with an E-value threshold of 1e-5. Simultaneously, BLASTP searches used known *OsHSP70* protein sequences from Arabidopsis thaliana as queries against the rice proteome database, with parameters including an E-value threshold of 1e-10, a word size of 6, and the BLOSUM62 substitution matrix.

Candidate sequences from both HMM and BLAST searches were combined, and duplicate entries were removed. All candidate *OsHSP70* proteins were further validated using SMART v9.0 (http://smart.embl-heidelverify and InterProScan v5.59–91.0 (http://www.ebi.ac.uk/interpro/) to verify the presence and integrity of conserved *OsHSP70* domains. Only sequences containing complete *OsHSP70* domains (both nucleotide-binding domain and substrate-binding domain) were kept for further analysis. Gene models with premature stop codons or incomplete domain structures were excluded, resulting in 32 validated *OsHSP70* genes.

### Physicochemical properties and subcellular localization prediction

Physicochemical properties of the 32 *OsHSP70* proteins, including molecular weight (MW), theoretical isoelectric point (pI), grand average of hydropathicity (GRAVY), instability index, and aliphatic index, were calculated using the ProtParam tool on the ExPASy server (https://web.expasy.org/protparam/). Proteins with instability index values below 40 were considered stable. Subcellular localization was predicted with WoLF PSORT v0.2 (https://wolfpsort.hgc.jp/) using plant-specific parameters. Results were visualized as heatmaps with TBtools v1.120, utilizing hierarchical clustering based on Euclidean distance and the complete linkage method.

### Chromosomal distribution and gene mapping

Chromosomal locations and gene coordinates were extracted from the rice genome GFF3 annotation file using TBtools v1.120. Physical positions were mapped and visualized using the Gene Location Visualize function in TBtools. Genes were considered as tandem clusters if they were located within 100 kb of each other on the same chromosome.

### Phylogenetic analysis and classification

Multiple sequence alignment of the 32 *OsHSP70* protein sequences was performed using ClustalW v2.1 with default parameters (gap opening penalty = 10, gap extension penalty = 0.2, and Gonnet protein weight matrix). Phylogenetic relationships were inferred using the Neighbor-Joining (NJ) method in MEGA X v10.2.6 with the following parameters: Poisson correction model, pairwise deletion for gap handling, and 1000 bootstrap replicates for statistical support. The resulting phylogenetic tree was edited and visualized using iTOL v6.7.4 (https://itol.embl.de/). Subfamily classification was determined based on phylogenetic clustering supported by bootstrap values ≥ 70%. Genes clustering together with strong bootstrap support were assigned to the same subfamily.

### Gene structure and motif analysis

Gene structure analysis was performed using the Gene Structure Display Server (GSDS) v2.0 (http://gsds.cbi.pku.edu.cn/) to visualize exon–intron organization. The CDS and genomic sequences were uploaded to generate gene structure diagrams, which show the relative positions and sizes of exons, introns, and untranslated regions (UTRs). Conserved protein motifs were identified using the MEME Suite v5.5.0 (http://meme-suite.org/tools/meme) with the following parameters: maximum number of motifs = 10, minimum motif width = 6 amino acids, maximum motif width = 70 amino acids, and E-value threshold of 1e-5. The identified motifs were functionally annotated using the NCBI Conserved Domain Database (CDD) and Pfam database.

### Gene duplication analysis and evolutionary rate calculation

Gene duplication events were identified using MCScanX v1.0 implemented in TBtools v1.120. Duplicate gene pairs were classified as tandem duplicates if located within 100 kb on the same chromosome, or as segmental duplicates if located on different chromosomes or > 100 kb apart on the same chromosome. For each duplicate gene pair, synonymous (Ks) and non-synonymous (Ka) substitution rates were calculated using the Yn00 method implemented in PAML v4.9 through TBtools. The Ka/Ks ratio was computed to determine the type of selective pressure: Ka/Ks < 1 indicates purifying selection, Ka/Ks = 1 indicates neutral evolution, and Ka/Ks > 1 indicates positive selection. Divergence time of duplicate gene pairs was estimated using the formula: T = Ks/(2λ), where λ = 6.56 × 10⁻⁹ substitutions per synonymous site per year for rice (Gaut et al. 1996).

### Protein–protein interaction network analysis

Protein–protein interaction (PPI) networks were constructed using the STRING database v12.0 (https://string-db.org/) with *Oryza sativa* as the target organism. The analysis included both direct (physical) and indirect (functional) associations with a confidence score threshold of 0.4 (medium confidence). Network visualization and analysis were performed using Cytoscape v3.9.1.

### Three-dimensional protein structure prediction

Representative *OsHSP70* protein structures were predicted using homology modeling through the SWISS-MODEL server (https://swissmodel.expasy.org/). The best templates were selected based on sequence identity and coverage. Model quality was assessed using GMQE (Global Model Quality Estimation) and QMEAN scoring functions.

### Plant materials and stress treatments for RNA expression analysis

#### Plant materials and growth conditions

A controlled pot experiment was conducted at King Abdullah University of Science and Technology, Thuwal, Saudi Arabia, during the summer season of 2023. Three rice cultivars were obtained from the Rice Research Department, Sakha Agricultural Research Station, Kafr El-Sheikh, Egypt. The experiment was designed to evaluate the comparative responses of these cultivars to multiple abiotic stress conditions at the seedling stage. Rice Sakha104 seeds were surface-sterilized with 3% (v/v) sodium hypochlorite solution for 10 min, rinsed thoroughly with sterile distilled water, and placed on moist filter paper for germination at room temperature (25 ± 2 °C). Pre-germinated seeds from each genotype were sown in plastic trays (30 × 20 × 10 cm) containing a sterilized mixture of field soil and farmyard manure (3:1, v/v). Seedlings were subsequently transferred to a controlled growth chamber maintained at 70% relative humidity, 24 ± 2 °C, with a 16 h photoperiod under photosynthetic photon flux density of 250–350 μmol m⁻^2^ s⁻^1^.

#### Abiotic stress treatments

Multiple abiotic stress treatments were applied to 14-day-old rice seedlings to evaluate *OsHSP70* gene expression responses. The following stress conditions were implemented: Heat Stress: Seedlings were exposed to elevated temperatures of 42 °C in the growth chamber for varying durations (15 min, 30 min, 1 h, 2 h, 3 h, and 4 h). Control plants were maintained at normal temperature (24 ± 2 °C). Salt Stress: Seedlings were treated with 150 mM NaCl solution applied to the soil medium. Sampling was conducted at 6 h, 24 h, and 48 h post-treatment. Control plants received distilled water. Cold Stress: Plants were subjected to low temperature stress at 4 °C for 6 h, 24 h, and 48 h. The temperature gradually reduced from 24 °C to 4 °C over 2 h to minimize shock effects. Drought Stress: Water withholding treatment was applied by stopping irrigation until soil moisture content reached 15% of field capacity. Leaf samples were collected when visible wilting symptoms appeared (typically 3–5 days post-treatment). Submergence Stress: Complete submergence was applied by flooding seedlings with water 5 cm above the plant canopy for 24 h and 48 h durations. All stress treatments were conducted in triplicate with at least 20 seedlings per treatment group. Environmental conditions in the growth chamber were maintained constant except for the specific stress parameter being tested.

#### RNA extraction and gene expression analysis

Leaf tissue samples (approximately 100 mg fresh weight) were collected from stressed and control plants at specified time points and immediately frozen in liquid nitrogen. Total RNA was extracted using TRIzol reagent (Invitrogen, USA) following the manufacturer protocol. RNA quality and concentration were assessed using a NanoDrop 2000 spectrophotometer (Thermo Scientific, USA) and agarose gel electrophoresis. Only RNA samples with A260/A280 ratios between 1.8–2.0 and clear ribosomal RNA bands were used for further analysis. First-strand cDNA synthesis was performed using 2 μg of total RNA with the SuperScript III First-Strand Synthesis System (Invitrogen, USA) with DNase I treatment to eliminate genomic DNA contamination. Gene-specific primers for the 32 *OsHSP70* genes were designed using Primer3Plus software (http://www.primer3plus.com/) with the following parameters: primer length 18–25 bp, melting temperature 58–62 °C, GC content 40–60%, and product size 150–200 bp. Primers were designed to target unique regions in the 3' portion of each gene coding sequence to ensure specificity. All primer sequences were verified using NCBI Primer-BLAST against the *Oryza sativa* genome to confirm single-target specificity. Prior to expression analysis, all primer pairs were validated by melting curve analysis (single peaks), agarose gel electrophoresis (single bands), standard curve analysis (efficiency 90–110%, R^2^ > 0.98), and no-template controls. Complete primer sequences, properties, and validation data are provided in Supplementary Table S1. Quantitative real-time PCR (qRT-PCR) was conducted using SYBR Green PCR Master Mix (Applied Biosystems, USA) on an ABI 7500 Real-Time PCR System. The PCR conditions included initial denaturation at 95 °C for 10 min, followed by 40 cycles of 95 °C for 15 s and 60 °C for 1 min. Melting curve analysis was performed to confirm primer specificity. The rice *Actin1* gene (*LOC_Os03g50885*) was used as an internal reference for normalization. All qRT-PCR experiments were performed with three independent biological replicates, where each biological replicate consisted of tissue pooled from at least 20 individual plants. This biological replication strategy captures true biological variation, which is the primary concern in gene expression studies per MIQE guidelines [35]. Technical variation was minimized through stringent quality control including RNA quality verification, validated primer efficiency (90–110%), automated liquid handling for cDNA synthesis, and single-operator execution. Expression levels were calculated using the 2^(-ΔΔCt) method (Livak and Schmittgen, 2001), with fold changes determined relative to control conditions. Statistical analysis was performed using one-way ANOVA followed by Tukey HSD test (P < 0.05, n = 3 biological replicates). Genes showing |log2(fold change)|≥ 1 and P < 0.05 were considered differentially expressed.

### Statistical analysis and data visualization

All statistical analyses were performed using R software v4.2.0. Correlation analyses were conducted using Pearson correlation coefficient. Heat maps were generated using the pheatmap package with hierarchical clustering. Statistical significance was set at P < 0.05 for all analyses. All figures and visualizations were created using TBtools v1.120, R software v4.2.0 with ggplot2 package, and Adobe Illustrator 2023. Color schemes were selected to be colorblind-friendly and publication-ready.

## Results

### Identification and characterization of *OsHSP70* gene family members in *Oryza sativa*

Genome-wide analysis of the *Oryza sativa* genome identified 32 *OsHSP70* family genes using combined HMM profiling and BLASTP searches. After validation through domain analysis with SMART and InterPro databases, all 32 candidates had complete and intact *OsHSP70* domains, confirming their status as genuine *OsHSP70* family members. The genes were systematically named *OsHSP70*−1 to *OsHSP70-32* based on their chromosomal locations. Comprehensive characterization of the physicochemical properties revealed considerable diversity among the *OsHSP70* proteins (Table S2). The protein lengths ranged from 216 amino acids (*LOC_Os12g38180.1*) to 903 amino acids (*LOC_Os02g48110.1*), with corresponding molecular weights varying from 24.33 kDa to 94.12 kDa. The theoretical isoelectric points (pI) showed broad distribution from 4.86 (*LOC_Os05g08840.1*) to 9.53 (*LOC_Os11g47760.3*), indicating diverse charge properties across family members.

The grand average of hydropathicity (GRAVY) values ranged from −0.471 to 0.059, with all proteins showing negative or near-neutral values, consistent with their hydrophilic nature as molecular chaperones. Stability analysis using the instability index revealed that all 32 *OsHSP70* proteins were predicted to be stable (instability index < 40), suggesting structural integrity under various cellular conditions. The aliphatic index varied between 75.62 and 98.23 (average 85.30), reflecting differences in thermostability among family members.

### Chromosomal localization and genomic distribution of *OsHSP70* genes

**Fig. 1:**
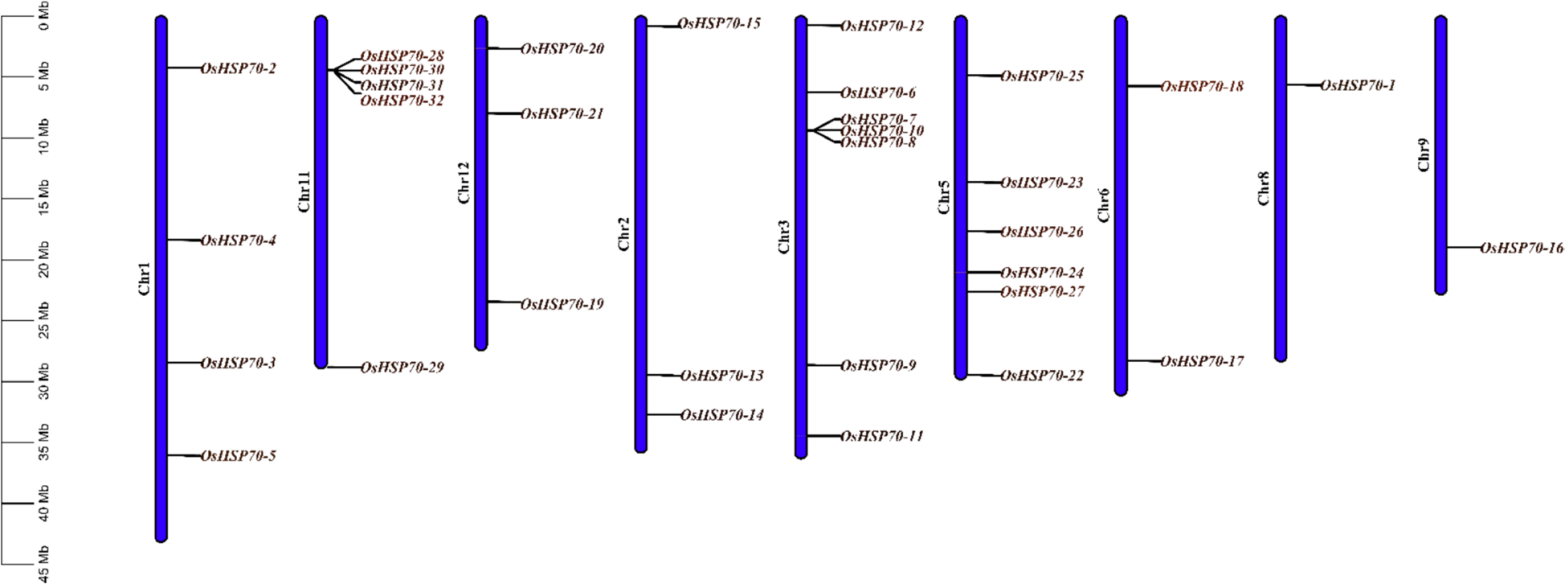
Chromosomal localization of *OsHSP70* genes in the rice genome. Physical map showing the distribution of 32 *OsHSP70* genes across 10 rice chromosomes (Chr1-Chr8, Chr10-Chr11). Chromosomes are represented as vertical blue bars scaled according to their physical length (Mb), with the scale shown on the left axis ranging from 0 to 45 Mb. Gene names are displayed on the right side of each chromosome at their corresponding genomic positions. Genes are color-coded with most genes shown in yellow/gold, while *OsHSP70-27* and *OsHSP70-8* are highlighted in red, potentially indicating unique structural or functional characteristics. The uneven distribution pattern reveals gene clustering on Chromosomes 2, 5, and 6, suggesting tandem duplication events, while dispersed genes on other chromosomes indicate segmental duplications. Chromosome 2 contains the highest density of *OsHSP70* genes with a prominent cluster in the proximal region (*OsHSP70-18*, *−20*, *−21*, *−23*, *−33*). Chromosome 6 harbors the most extensive tandem cluster with four genes (*OsHSP70-24*, *−26*, *−27*, *−31*) positioned in close proximity around 15–20 Mb. No *OsHSP70* genes were identified on Chromosomes 9 and 12, indicating chromosome-specific gene loss or absence of ancestral *OsHSP70* loci on these chromosomes. The distribution pattern reflects the evolutionary dynamics of the *OsHSP70* family, shaped by tandem duplications (creating local clusters), segmental duplications (dispersing genes across chromosomes), and potential gene loss events. This genomic organization provides insights into the mechanisms underlying *OsHSP70* gene family expansion in rice and has implications for understanding stress tolerance mechanisms and facilitating marker-assisted breeding strategies targeting *OsHSP70*-mediated stress responses

To understand the genomic organization and distribution patterns of *OsHSP70* genes across the rice genome, chromosomal localization analysis was performed using genomic coordinates from the rice reference genome (Fig. 1). The analysis revealed that 32 *OsHSP70* genes were distributed unevenly across 10 of the 12 rice chromosomes, with no *OsHSP70* genes identified on chromosomes 9 and 12. This non-random distribution pattern suggests that *OsHSP70* genes may have undergone preferential retention or loss on specific chromosomes during rice genome evolution. Chromosome 1 harbored the highest number of *OsHSP70* genes with four members (*OsHSP70-4*, *OsHSP70-6*, *OsHSP70-8*, and *OsHSP70-5*), distributed across different regions of the chromosome. *OsHSP70-8* was located in the sub telomeric region at approximately 34 Mb, while *OsHSP70-4* was positioned at around 26 Mb, *OsHSP70-6* at approximately 30 Mb, and *OsHSP70-5* near 42 Mb. The dispersed distribution of these genes along Chromosome 1 suggests they likely arose through segmental duplication events rather than tandem duplications. Chromosome 2 contained five *OsHSP70* genes (*OsHSP70-2*, *OsHSP70-18*, *OsHSP70-20*, *OsHSP70-21*, *OsHSP70-23*, and *OsHSP70-33*), making it one of the most gene-dense chromosomes for this family. Notably, several genes on Chromosome 2 appeared clustered in the proximal region between 0–10 Mb (*OsHSP70-18*, *OsHSP70-20*, *OsHSP70-21*, *OsHSP70-23*, and *OsHSP70-33*), suggesting this region may represent a hotspot for tandem duplication events that contributed to local HSP70 gene expansion.

Chromosome 3 contained four *OsHSP70* genes (*OsHSP70-19*, *OsHSP70-21*, *OsHSP70-30*, and *OsHSP70-29*), distributed across the chromosome length from approximately 20 Mb to 30 Mb. The spacing between these genes indicates they are not tandemly arrayed but rather represent dispersed duplications. Chromosome 4 harbored two genes (*OsHSP70-13* and *OsHSP70-14*) located in relatively proximal positions around 30–35 Mb, suggesting possible ancestral tandem duplication followed by chromosomal rearrangement. Chromosome 5 contained three *OsHSP70* genes (*OsHSP70-15*, *OsHSP70-12*, *OsHSP70-4*, *OsHSP70-20*, and *OsHSP70-23*), with *OsHSP70-4*, *OsHSP70-20*, and *OsHSP70-23* forming a cluster in the proximal region, while *OsHSP70-15* and *OsHSP70-12* were positioned more distally. This pattern suggests a combination of tandem and segmental duplications contributed to the *OsHSP70* complement on Chromosome 5.

Chromosome 6 showed a notable concentration of *OsHSP70* genes with six members (*OsHSP70-23*, *OsHSP70-26*, *OsHSP70-32*, *OsHSP70-24*, *OsHSP70-27*, and *OsHSP70-31*). The clustering of *OsHSP70-24*, *OsHSP70-26*, *OsHSP70-27*, and *OsHSP70-31* in the middle region of the chromosome (approximately 15–20 Mb) represents the most prominent tandem gene cluster in the entire *OsHSP70* family, suggesting that tandem duplication has been a major driver of *OsHSP70* expansion on this chromosome. *OsHSP70-27* is distinctly marked in red on the figure, potentially indicating a unique structural or functional characteristic that distinguishes it from other clustered members. Chromosome 7 contained three genes (*OsHSP70-17*, *OsHSP70-16*, and *OsHSP70-1*), distributed along the chromosome from approximately 20 Mb to the distal end. The dispersed nature of these genes suggests segmental duplication or chromosomal rearrangement events. Chromosome 8 harbored only a single *OsHSP70* gene (*OsHSP70-16*), located in the distal region of the chromosome, making it one of the least *OsHSP70*-dense chromosomes. The singleton status of this gene suggests either gene loss events on Chromosome 8 or that this represents an ancient *OsHSP70* locus that did not undergo local expansion. Chromosome 10 contained two genes (*OsHSP70-14* and *OsHSP70-13*) positioned in the central region around 30–35 Mb, potentially representing a small tandem duplication event. Chromosome 11 also contained two *OsHSP70* genes (*OsHSP70-9* and *OsHSP70-31*), with *OsHSP70-9* located proximally around 25 Mb and *OsHSP70-31* positioned more distally, indicating dispersed distribution rather than tandem arrangement. The chromosomal distribution analysis revealed several important features of *OsHSP70* genome organization. First, the presence of gene clusters on Chromosomes 2, 5, and 6 indicates that tandem duplication has been a significant mechanism for *OsHSP70* gene family expansion. Tandem duplications typically generate closely related paralogs that may initially have redundant functions but can subsequently diverge through sub functionalization or neofunctionalization. The most prominent cluster on Chromosome 6, containing four genes within a relatively short genomic span, represents an excellent model for studying the evolutionary fate of tandemly duplicated *OsHSP70* genes. Second, the presence of dispersed genes on multiple chromosomes suggests that segmental duplications, including whole-genome or large-scale duplication events, have also contributed to *OsHSP70* gene family expansion in rice. The absence of *OsHSP70* genes on Chromosomes 9 and 12 is noteworthy and suggests either complete gene loss from these chromosomes or that ancestral *OsHSP70* genes were never present on these chromosomal segments. Comparative genomic analysis with other grass species could help determine whether this distribution pattern is rice-specific or reflects broader patterns in cereal evolution. The uneven distribution of *OsHSP70* genes, with some chromosomes containing six genes while others contain none, contrasts with the more uniform distribution patterns observed for some housekeeping gene families and may reflect the dynamic evolutionary history of the *OsHSP70* family in response to environmental selection pressures. Overall, the chromosomal distribution analysis reveals that the rice *OsHSP70* gene family has been shaped by multiple duplication mechanisms operating over different evolutionary timescales. The combination of tandem duplications creating local gene clusters and segmental duplications distributing genes across multiple chromosomes has generated the structural foundation for functional diversification. This genomic organization provides rice with a flexible *OsHSP70* chaperone system distributed across multiple chromosomes, potentially ensuring that at least some *OsHSP70* genes are retained even if chromosomal rearrangements or deletions occur during evolution or breeding. The non-random distribution pattern also suggests that specific chromosomal contexts may be more permissive for *OsHSP70* gene evolution and function, a hypothesis that could be tested through comparative genomic and functional studies.

### Phylogenetic relationships and subfamily classification

To investigate the evolutionary relationships and functional diversification of *OsHSP70* genes, a comprehensive phylogenetic analysis was performed using the neighbor-joining method with 1000 bootstrap replicates (Fig. 5). The phylogenetic tree revealed that the 32 *OsHSP70* proteins clustered into six distinct subfamilies (I-VI), supported by high bootstrap values (> 70%), indicating robust evolutionary relationships and suggesting functional divergence within the rice *HSP70* family. Subfamily I (brown/tan) comprised six members, including *OsHSP70-16*, *OsHSP70-18*, *OsHSP70-19*, *OsHSP70-20*, *OsHSP70-26*, and *OsHSP70-27*. This group showed relatively shorter branch lengths, suggesting recent divergence or strong purifying selection. The compact gene structures and shorter protein lengths (400–500 amino acids) observed in several members of this subfamily, particularly *OsHSP70-18*, *OsHSP70-19*, and *OsHSP70-20*, suggest they may represent a specialized subset of *HSP70*s with streamlined chaperone functions, possibly localized to specific organelles such as chloroplasts or mitochondria. Subfamily II (red) included five members: *OsHSP70-2*, *OsHSP70-3*, *OsHSP70-17*, *OsHSP70-22*, and *OsHSP70-30*. The longer branch lengths in this subfamily indicate greater sequence divergence, suggesting either relaxed selection pressure or adaptive evolution to acquire specialized functions. The presence of both full-length proteins such as *OsHSP70-2* and *OsHSP70-30*, alongside a shorter variant *OsHSP70-17*, within this clade suggests functional diversification through domain loss or gain events. Subfamily III (blue/teal) was the second-largest group, containing seven members: *OsHSP70-1*, *OsHSP70-5*, *OsHSP70-13*, *OsHSP70-19*, *OsHSP70-25*, *OsHSP70-29*, and *OsHSP70-31*. This subfamily displayed moderate branch lengths and included *OsHSP70-13*, which possesses the longest gene structure (~ 7000 bp) in the entire family. The presence of both highly complex gene structures like *OsHSP70-13* and moderately complex structures suggests this subfamily may have undergone extensive structural remodeling during evolution, potentially acquiring additional regulatory elements or alternative splicing capabilities. Subfamily IV (purple) represented the largest and most diverse subfamily, comprising 12 members: *OsHSP70-4*, *OsHSP70-6*, *OsHSP70-7*, *OsHSP70-8*, *OsHSP70-9*, *OsHSP70-10*, *OsHSP70-11*, *OsHSP70-12*, *OsHSP70-15*, *OsHSP70-24*, *OsHSP70-32*, and *OsHSP70-82*. The large size of this subfamily and the star-like topology with short internal branches suggest a recent expansion, possibly through tandem or segmental duplications. Many members of this subfamily contained the complete 10-motif set and displayed typical cytosolic *OsHSP70* characteristics, suggesting they may represent the core constitutive and stress-inducible cytosolic chaperones essential for general protein homeostasis. Subfamily V (green) consisted of four members: *OsHSP70-14*, *OsHSP70-33*, *OsHSP70-41*, *OsHSP70-42*, and *OsHSP70-43*. This well-supported monophyletic group showed distinct separation from other subfamilies, indicating early divergence from a common ancestral HSP70 gene. The evolutionary distance suggests these proteins may have evolved specialized functions distinct from the canonical *OsHSP70* chaperone activities. Subfamily VI (yellow/tan) comprised only two members, *HSP70-8* and *HSP70-9*, forming a small but distinct clade. This subfamily small size and phylogenetic position suggest either gene loss events in rice or highly specialized functions that limit gene expansion. The phylogenetic distribution revealed several evolutionary patterns consistent with gene family expansion mechanisms. The clustering of multiple *OsHSP70* genes within subfamilies with short branch lengths, particularly in Subfamily IV, suggests recent tandem or segmental duplication events. These duplications likely contributed to the functional diversification of *OsHSP70* proteins, enabling the plant to develop specialized chaperone systems for different cellular compartments, developmental stages, and stress conditions. The star-like topology observed in Subfamily IV, where multiple genes radiate from a common node with minimal branch length differences, is characteristic of rapid gene expansion through duplication events. Such expansion patterns have been documented in other stress-responsive gene families and are thought to provide evolutionary raw material for functional innovation and adaptation to diverse environmental challenges.

Comparison with *HSP70* families from other plant species provides important context for understanding the evolutionary dynamics of this gene family in rice. Previous studies in maize (*Zea mays*) identified 28 *HSP70* genes, while wheat (*Triticum aestivum*) contains approximately 50 *HSP70* genes across its three subgenomes, and the dicot model plant *Arabidopsis thaliana* possesses 18 *HSP70* genes. The moderate expansion in rice (32 genes) relative to diploid dicots and other monocots suggests balanced evolutionary pressures maintaining sufficient functional diversity while avoiding excessive redundancy. The phylogenetic structure also suggests that several rice-specific duplications occurred after the monocot-dicot split, contributing to lineage-specific adaptations. Wheat larger *HSP70* complement reflects its polyploid nature rather than functional expansion, as the three subgenomes each contain similar numbers of *HSP70* genes to rice. Maize, despite having a similar genome size to rice, has fewer *HSP70* genes, suggesting different evolutionary strategies in these two major cereal crops. The branch length patterns across the phylogenetic tree provide insights into selection pressures acting on different *OsHSP70* subfamilies. Subfamilies with shorter terminal branch lengths, such as Subfamily I and portions of Subfamily IV, indicate strong purifying selection maintaining conserved sequences, likely reflecting their essential housekeeping functions in protein folding and stress responses. In contrast, Subfamily II members exhibit longer branch lengths, suggesting either relaxed purifying selection allowing neutral mutations to accumulate, or positive selection driving functional divergence. The ratio of synonymous to non-synonymous substitution rates would further elucidate whether these proteins are under neutral evolution or adaptive selection. The intermediate branch lengths observed in Subfamily III suggest a balance between conservation of core chaperone functions and acquisition of specialized features, consistent with the structural diversity observed in this group.

**Fig. 2:**
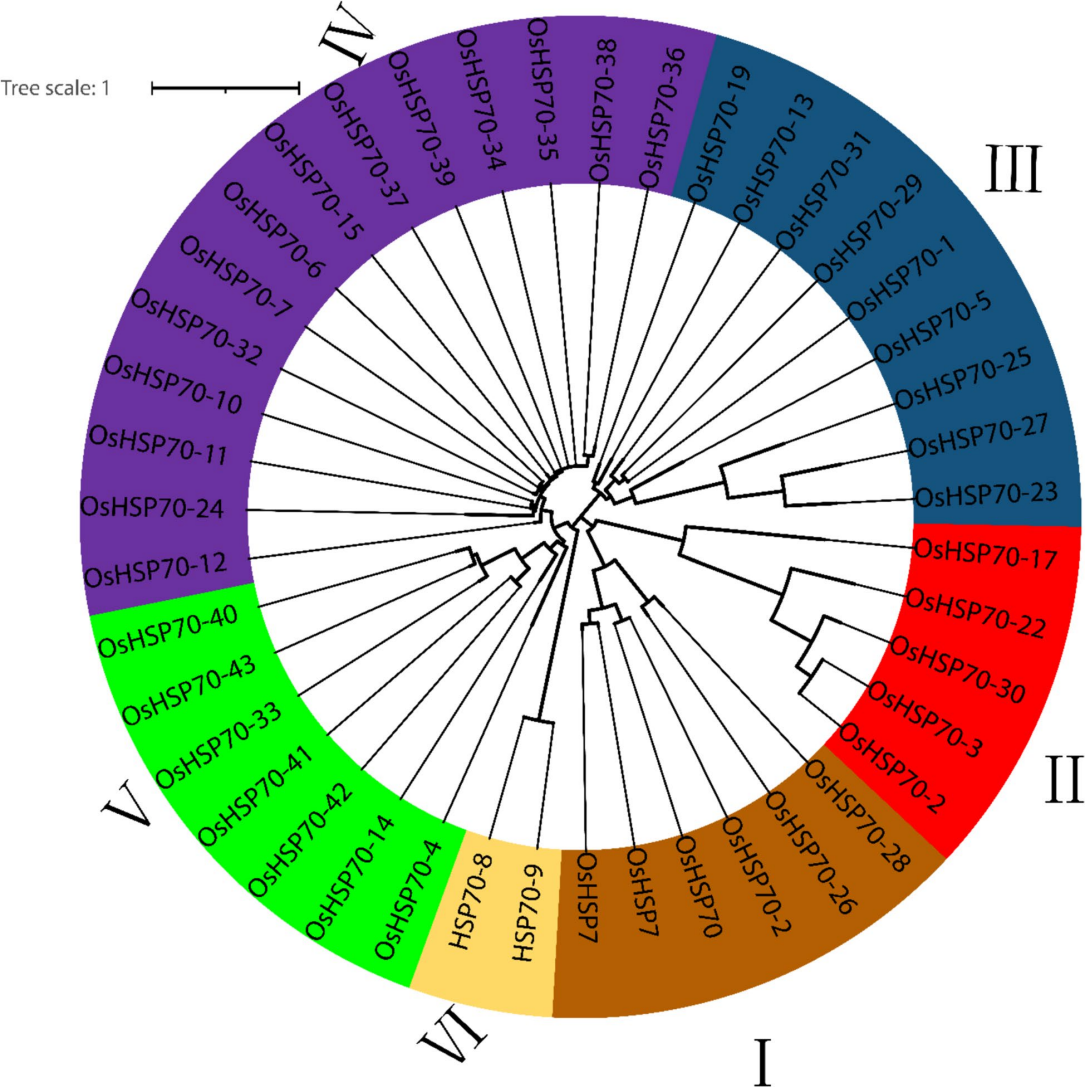
Phylogenetic analysis and subfamily classification of *OsHSP70* proteins. Circular phylogenetic tree constructed from 32 rice *OsHSP70* protein sequences using the neighbor-joining method with 1000 bootstrap replicates in MEGA11 software. The tree reveals six distinct evolutionary subfamilies (I-VI) indicated by different colors. Subfamily I (brown/tan) contains six members including *OsHSP70-16*, *−18*, *−19*, *−20*, *−26*, and *−27*, characterized by shorter proteins with compact gene structures suggesting specialized organellar functions. Subfamily II (red) includes five members (*OsHSP70-2*, *−3*, *−17*, *−22*, *−30*) and exhibits longer branch lengths indicating greater sequence divergence and potential functional specialization. Subfamily III (blue/teal) comprises seven members (*OsHSP70-1*, *−5*, *−13*, *−19*, *−25*, *−29*, *−31*) and contains the most structurally complex gene (*OsHSP70-13* with approximately 7000 bp), suggesting acquisition of additional regulatory elements. Subfamily IV (purple) is the largest with 12 members (*OsHSP70-4*, *−6*, *−7*, *−8*, *−9*, *−10*, *−11*, *−12*, *−15*, *−24*, *−32*, *−82*), displaying a star-like topology that indicates recent expansion through gene duplication and likely represents core cytosolic *HSP70s*. Subfamily V (green) includes four members (*OsHSP70-14*, *−33*, *−41*, *−42*, *−43*) forming a distinct clade suggesting early divergence and specialized functions. Subfamily VI (yellow/tan) contains only two members (*HSP70-8*, *HSP70-9*), indicating either gene loss or highly specialized functions. The tree scale bar represents the number of amino acid substitutions per site (scale = 1), with branch lengths proportional to evolutionary distance. The clustering patterns reveal recent duplication events (short internal branches in Subfamily IV), purifying selection (short terminal branches), and functional diversification (long branches in Subfamily II)

The evolutionary analysis indicates that *OsHSP70* proteins have undergone both purifying and positive selection at different evolutionary time points, resulting in a family that maintains core conserved chaperone functions while acquiring specialized roles. Ancient duplications gave rise to the major subfamilies, each of which subsequently underwent lineage-specific expansions and functional refinements. More recent duplications, particularly evident in Subfamily IV, have generated closely related paralogs that may exhibit subtle functional differences in expression patterns, subcellular localization, substrate specificity, or co-chaperone interactions. This evolutionary flexibility has likely been crucial for rice adaptation to diverse environmental conditions, including heat stress, drought, salinity, and pathogen challenges that rice encounters in its natural wetland habitats and diverse agricultural settings ranging from irrigated lowlands to rain-fed uplands and deepwater environments. The presence of multiple *OsHSP70* subfamilies with distinct evolutionary histories suggests that rice has evolved a sophisticated multi-layered chaperone system capable of responding to the complex and variable stress conditions characteristic of rice cultivation worldwide (Fig. 2).

### Subcellular localization predictions

**Fig. 3:**
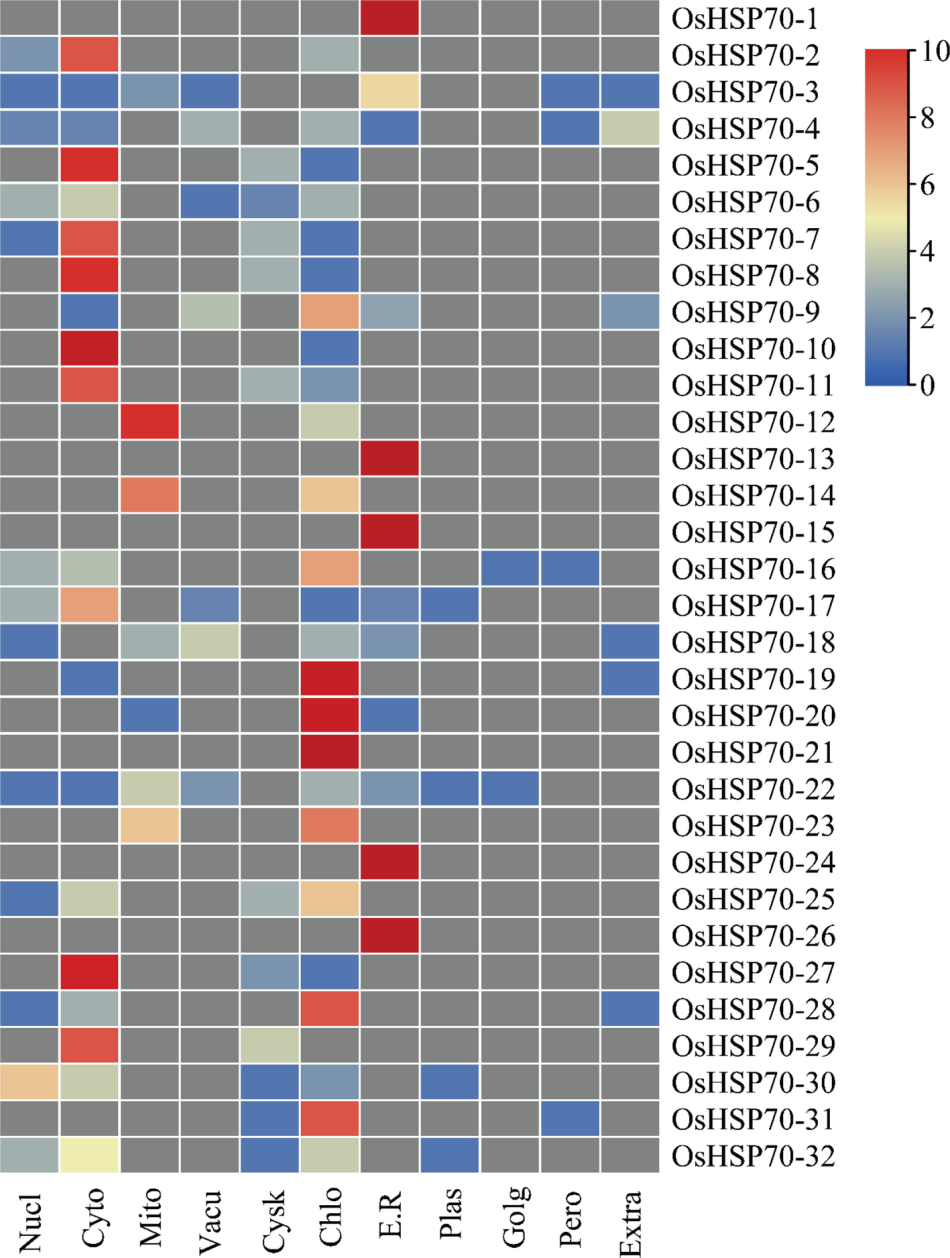
Subcellular localization prediction and expression patterns of rice *OsHSP70* proteins. The heatmap displays the predicted subcellular localization scores for 32 *OsHSP70* proteins across 11 different cellular compartments. Rows represent individual *OsHSP70* proteins (*OsHSP70-1* to *OsHSP70-32*), while columns indicate different subcellular compartments: Nucl (nucleus), Cyto (cytoplasm), Mito (mitochondria), Vacu (vacuole), Cysk (cytoskeleton), Chlo (chloroplast), E.R. (endoplasmic reticulum), Plas (plasma membrane), Golg (Golgi apparatus), Pero (peroxisome), and Extra (extracellular). Color intensity represents WoLF PSORT prediction scores, with red indicating high confidence scores (10), blue indicating low scores (0), and intermediate colors (orange, yellow, light blue) representing moderate confidence levels. Gray cells indicate neutral or baseline prediction values. Proteins were hierarchically clustered using Euclidean distance and complete linkage method to group proteins with similar localization patterns. Predictions were generated using WoLF PSORT v0.2 with plant-specific parameters, and the heatmap was visualized using TBtools v1.120

Subcellular localization prediction analysis was conducted for 32 *OsHSP70* proteins using WoLF PSORT v0.2 with plant-specific parameters to determine their predicted intracellular targeting and distribution patterns (Fig. 3 and Table S3). The analysis evaluated the likelihood of protein localization across 11 different subcellular compartments, including nucleus, cytoplasm, mitochondria, vacuole, cytoskeleton, chloroplast, endoplasmic reticulum, plasma membrane, Golgi apparatus, peroxisome, and extracellular space. The prediction scores, ranging from 0 to 10, were visualized as a heatmap using hierarchical clustering based on Euclidean distance and complete linkage method, revealing diverse subcellular localizations that reflect the functional specialization of *OsHSP70* proteins in various cellular processes. The heatmap analysis revealed distinct localization patterns across the *OsHSP70* family. Several proteins showed strong prediction scores (indicated by red coloration) for specific compartments, suggesting high confidence in their subcellular targeting. For example, *OsHSP70-1, OsHSP70-13, OsHSP70-24*, and *OsHSP70-26* exhibited the highest prediction scores for endoplasmic reticulum localization, indicating their likely roles in ER protein quality control and the unfolded protein response. *OsHSP70-15* showed strong chloroplast targeting prediction, highlighting its potential function in photosynthetic apparatus protection and chloroplast stress tolerance. Cytoplasmic localization was predicted with moderate to high confidence for multiple proteins, including *OsHSP70-2*, *OsHSP70-7, OsHSP70-8, OsHSP70-9, OsHSP70-10*, and *OsHSP70-11*, as indicated by red to orange coloration in the cytoplasm column. These cytosolic *OsHSP70* proteins likely function in general protein folding, cytoplasmic stress response, and maintenance of cellular protein homeostasis under various environmental stresses. Several proteins displayed blue coloration (low prediction scores) for most compartments with specific red peaks, suggesting highly specialized subcellular targeting. For instance, *OsHSP70-3, OsHSP70-18,* and *OsHSP70-22* showed strong cytoplasmic predictions, while *OsHSP70-5, OsHSP70-19*, *OsHSP70-20*, and *OsHSP70-21* exhibited notable chloroplast localization scores. The mitochondrial column revealed high prediction scores for *OsHSP70-19*, indicating its potential role in mitochondrial protein quality control and respiratory chain protection. Interestingly, some proteins exhibited intermediate prediction scores (yellow to orange coloration) across multiple compartments, suggesting potential dual localization or context-dependent targeting mechanisms. For example, *OsHSP70-4* and *OsHSP70-6* showed moderate scores for both cytoplasm and chloroplast, indicating possible shuttle functions between these compartments or tissue-specific localization patterns. Nuclear localization predictions were generally lower across the family, with only a few proteins showing moderate scores, suggesting that most *OsHSP70* proteins primarily function in non-nuclear compartments. Similarly, predictions for Golgi apparatus, peroxisome, and extracellular space were relatively low for most proteins, with only occasional moderate scores. The hierarchical clustering pattern revealed groupings of proteins with similar subcellular localization profiles, suggesting potential functional relationships or evolutionary origins. Proteins clustered together in the heatmap may share similar targeting signals, chaperone specificities, or stress response functions within the same cellular compartment. The diverse subcellular distribution patterns demonstrated by the heatmap analysis illustrate that *OsHSP70* family members have evolved specialized roles across various cellular compartments, enabling comprehensive cellular protection under different stress conditions. This compartment-specific distribution reflects the evolutionary adaptation of rice *OsHSP70* proteins to sustain protein homeostasis throughout the cell, ensuring proper protein folding, assembly, and degradation in organelle-specific environments. The presence of *OsHSP70* proteins in multiple compartments, particularly in metabolically active sites such as chloroplasts, mitochondria, and the ER, underscores their critical importance in maintaining cellular function during both normal growth and stress responses.

### HSP70 protein domain and motif analysis

**Fig. 4:**
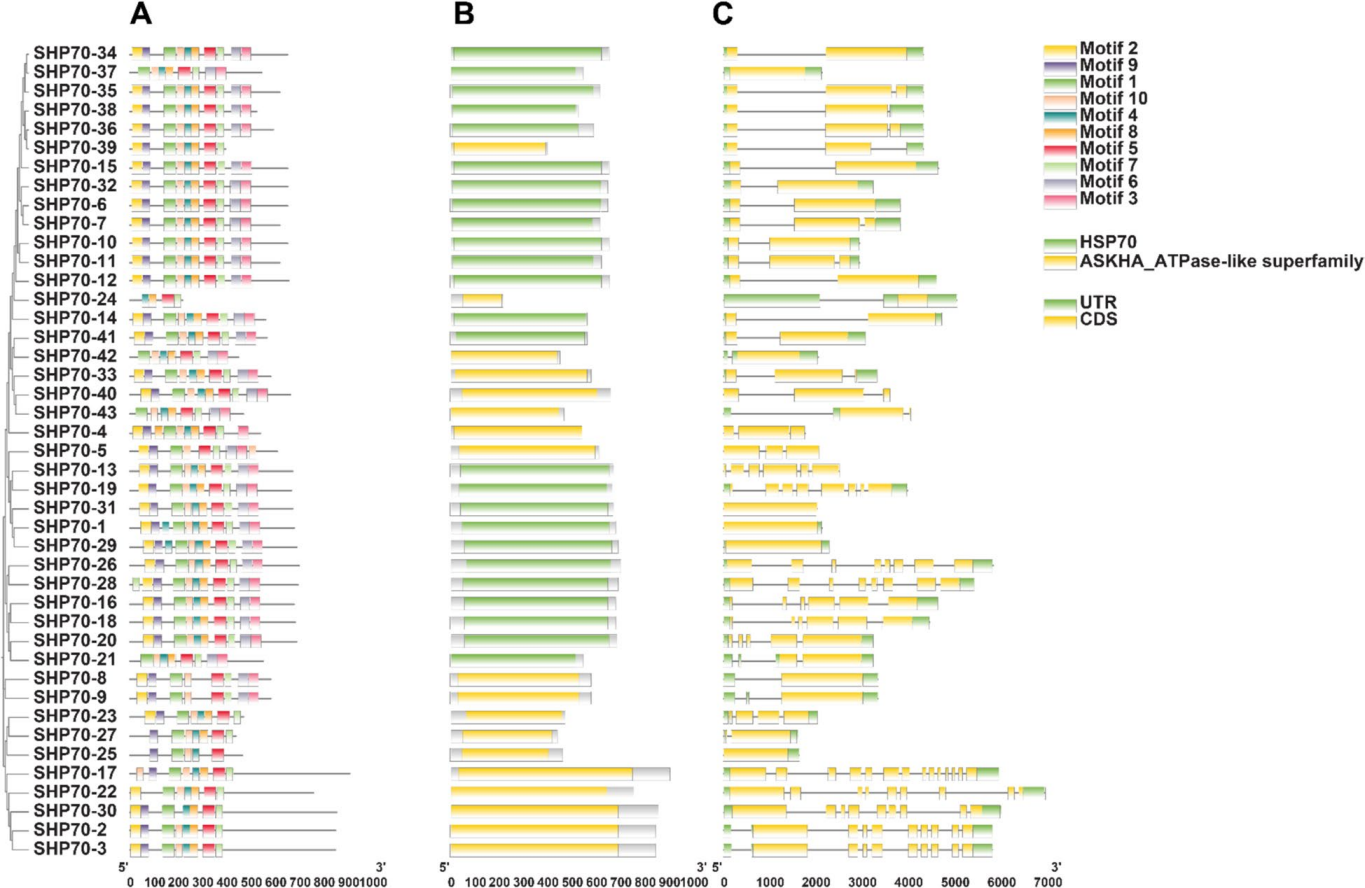
Integrated structural analysis of rice *OsHSP70* gene family showing gene organization, functional domain architecture, and conserved motif distribution. **A** Gene structure organization: Exon–intron arrangements for 32 *OsHSP70* genes displayed to scale (0–7000 bp). Yellow boxes represent protein-coding sequences (CDS), white boxes indicate 5' and 3' untranslated regions (UTR), and black lines represent introns. The variable gene structures reflect different evolutionary trajectories and may influence mRNA stability, translation efficiency, and stress-responsive expression regulation. Complex multi-intron genes (*OsHSP70-13*, *OsHSP70-23*, *OsHSP70-17*) may undergo alternative splicing, while compact intron-poor genes (*OsHSP70-18*, *OsHSP70-19*, *OsHSP70-20*) may enable rapid stress-induced expression. **B** Functional domain architecture: Distribution of the ASKHA_ATPase-like superfamily domain (yellow/gold) and *OsHSP70* domain (green) across all 32 proteins (0–900 amino acids scale). The ASKHA_ATPase-like domain encompasses the N-terminal nucleotide-binding domain (NBD) responsible for ATP binding, hydrolysis, and the conformational changes that drive the chaperone cycle. Extended C-terminal regions beyond this domain in proteins like *OsHSP70-13*, *OsHSP70-25*, and *OsHSP70-26* likely contain additional regulatory elements, subcellular targeting signals, or protein–protein interaction motifs that modulate *OsHSP70* activity and localization. **C** Conserved motif distribution and functional architecture: Ten distinct conserved motifs identified by MEME analysis (E-values: 1.10 × 10⁻⁷^2^ to 2.46 × 10⁻^1^⁹^4^) are displayed across 32 *OsHSP70* proteins (0–1000 amino acids scale). Motif colors and their functional roles are as follows: N-terminal NBD/ATPase region (Motifs 7–9-1): Motif 7 (light green), Motif 9 (purple), and Motif 1 (dark green) form the highly conserved core of the nucleotide-binding domain. These motifs contain critical residues for ATP binding (Walker A and B motifs), Mg^2^⁺ coordination, and nucleotide hydrolysis. The 7–9-1 arrangement is nearly universal across the family, reflecting the absolute requirement for ATP-dependent chaperone activity. Central SBD region (Motifs 3–6-4–2–5): Motif 3 (pink), Motif 6 (gray), Motif 4 (teal), Motif 2 (yellow), and Motif 5 (red) constitute the substrate-binding domain responsible for recognizing hydrophobic patches on misfolded or nascent polypeptides. The characteristic 3–6-4–2–5 arrangement forms the β-sandwich structure and α-helical lid that together create the substrate-binding pocket. Motif 3 universal presence indicates its critical role in substrate recognition, while variations in motifs 2, 4, and 5 may determine substrate specificity and binding affinity. C-terminal regulatory region (Motifs 8–10): Motif 8 (orange) and Motif 10 (light orange/peach) show variable distribution and likely mediate protein–protein interactions with co-chaperones (HSP40/J-proteins, nucleotide exchange factors), regulate the rate of substrate release, or contain the EEVD motif for tetratricopeptide repeat (TPR) domain interactions. Motif 10 restricted distribution to 50% of proteins suggests subfamily-specific regulatory mechanisms or specialized co-chaperone requirements. Proteins are ordered identically across all three panels to facilitate direct comparison between gene structure, domain organization, and motif architecture. This integrated view reveals how genomic organization (**A**) translates to domain structure (**B**) and functional motif composition (**C**), illustrating the evolutionary plasticity and functional diversification of the rice *HSP70* chaperone system

Comprehensive structural analysis of the 32 *OsHSP70* genes revealed diverse gene structures, conserved domain architecture, and modular motif organization characteristic of the *HSP70* family (Fig. 4, Table S4). Gene structure analysis revealed considerable diversity in exon–intron organization (Fig. 4A). Gene lengths varied dramatically from compact structures under 1,000 bp (*OsHSP70-6*, *OsHSP70-18*, *OsHSP70-19*, *OsHSP70-20*) to highly complex genes spanning approximately 7,000 bp (*OsHSP70-13*). Most *OsHSP70* genes contained 1–3 introns interrupting the coding sequences (CDS, shown in yellow), with intron positions generally conserved among closely related members. Several genes, including *OsHSP70-23*, *OsHSP70-17*, and *OsHSP70-13*, displayed more complex intron–exon architectures with multiple introns, suggesting potential for alternative splicing or enhanced post-transcriptional regulation. The coding sequences were flanked by 5' and 3' untranslated regions (UTRs, shown in white) of variable lengths. Interestingly, gene structure complexity did not always correlate directly with protein length, indicating diverse evolutionary pathways within the family. Domain architecture analysis confirmed the presence of the characteristic ASKHA_ATPase-like superfamily domain (shown in yellow/gold) across all 32 sequences (Fig. 4B), validating their identity as bona fide *OsHSP70* family members. The domain extended across the N-terminal to central regions, with lengths varying from approximately 200 amino acids in compact proteins (*OsHSP70-6*, *OsHSP70-20*) to over 800 amino acids in larger proteins (*OsHSP70-13*, *OsHSP70-25*, *OsHSP70-2*). Some proteins displayed extended C-terminal regions beyond the annotated domain, suggesting additional regulatory or interaction modules. MEME analysis identified 10 distinct conserved motifs with varying distribution patterns and statistical significance (E-values ranging from 1.10 × 10⁻⁷^2^ to 2.46 × 10⁻^1^⁹^4^) (Fig. 4C). The spatial organization revealed a highly conserved modular architecture. Motifs 7 (light green), 9 (purple), and 1 (dark green) formed a conserved N-terminal trio present in nearly all family members, corresponding to the nucleotide-binding domain (NBD). This characteristic 7–9-1 arrangement is essential for ATP binding and hydrolysis. The central protein region contained motifs 3 (pink), 6 (gray), 4 (teal), 2 (yellow), and 5 (red), typically appearing in order 3–6-4–2–5, which constitute the substrate-binding domain (SBD) responsible for recognizing and binding misfolded substrates. Motif 3 showed particularly high conservation across all proteins. The C-terminal region featured motifs 8 (orange) and 10 (light orange/peach) with markedly different distribution patterns. Motif 8 was present in approximately 60% of the proteins (*OsHSP70-1*, *OsHSP70-2*, *OsHSP70-7* through *OsHSP70-12*, *OsHSP70-14*, *OsHSP70-16*, *OsHSP70-21* through *OsHSP70-23*, *OsHSP70-27*, *OsHSP70-82*, *OsHSP70-29*, *OsHSP70-30*), likely playing roles in regulatory functions or co-chaperone interactions. Motif 10 showed the most restricted distribution, appearing in only 16 proteins (*OsHSP70-1*, *OsHSP70-2*, *OsHSP70-4*, *OsHSP70-7* through *OsHSP70-11*, *OsHSP70-14*, *OsHSP70-21* through *OsHSP70-23*, *OsHSP70-30*, *OsHSP70-31*), suggesting functional subspecialization. Protein length variation was substantial. The longest proteins (*OsHSP70-13* at ~ 900 amino acids, *OsHSP70-25* at ~ 850 amino acids) contained extended C-terminal regions beyond the conserved motif array. The shortest proteins (*OsHSP70-17*, *OsHSP70-18*, *OsHSP70-19*, *OsHSP70-20* at 400–450 amino acids) contained primarily core N-terminal and central motifs, representing minimal functional HSP70 units. Most full-length proteins ranged from 550–650 amino acids and contained 7–9 motifs.

The correlation between gene structure, domain organization, and motif composition revealed distinct evolutionary patterns. Proteins with complete motif sets (including motifs 8 and 10) generally originated from genes with more complex structures, while minimal *OsHSP70* units typically derived from compact, intron-poor genes. This modular organization suggests that *OsHSP70* genes evolved through domain duplication, deletion, intron gain/loss, and recombination events, enabling functional diversification while maintaining core chaperone activities. The variable motif compositions, combined with differences in gene structure and subcellular targeting sequences, likely contribute to substrate specificity, subcellular localization, co-chaperone interactions, and stress-responsive expression patterns, enabling *OsHSP70* proteins to provide comprehensive cellular protection under diverse environmental conditions.

### Gene duplication analysis and evolutionary rates

Analysis of gene duplication events identified both tandem and segmental duplications contributing to *OsHSP70* gene family expansion in rice. A total of 18 duplicate gene pairs were detected, including 12 segmental duplicates and 6 tandem duplicates, indicating that segmental duplication was the predominant mechanism of gene family expansion. Ka/Ks ratio analysis revealed that most duplicate gene pairs (83%, n = 15) exhibited Ka/Ks ratios significantly less than 1 (range: 0.12–0.89), indicating strong purifying selection and highlighting evolutionary pressure to maintain protein function (Table S5). This suggests the critical importance of HSP70 proteins in rice biology and stress tolerance. Three gene pairs showed Ka/Ks ratios close to or slightly exceeding 1 (*LOC_Os01g33360.1–LOC_Os05g35400.1, LOC_Os03g16880.1–LOC_Os11g08445.1,* and *LOC_Os01g62290.1–LOC_Os05g30480.1*), potentially indicating episodes of relaxed selection or functional divergence following duplication. Divergence time estimation revealed a wide range of duplication events, from recent duplications less than 1 million years ago (Mya) to ancient events exceeding 60 Mya. The most recent duplication (*LOC_Os05g35400.1–LOC_Os08g09770.1*) occurred approximately 0.98 Mya, while the most ancient (*LOC_Os01g08560.1–LOC_Os03g11910.1*) was estimated at 64.75 Mya. This temporal distribution suggests both recent species-specific expansions and ancient duplications, possibly associated with whole-genome duplication events in the rice lineage.

### Protein–protein interaction network analysis

**Fig. 5:**
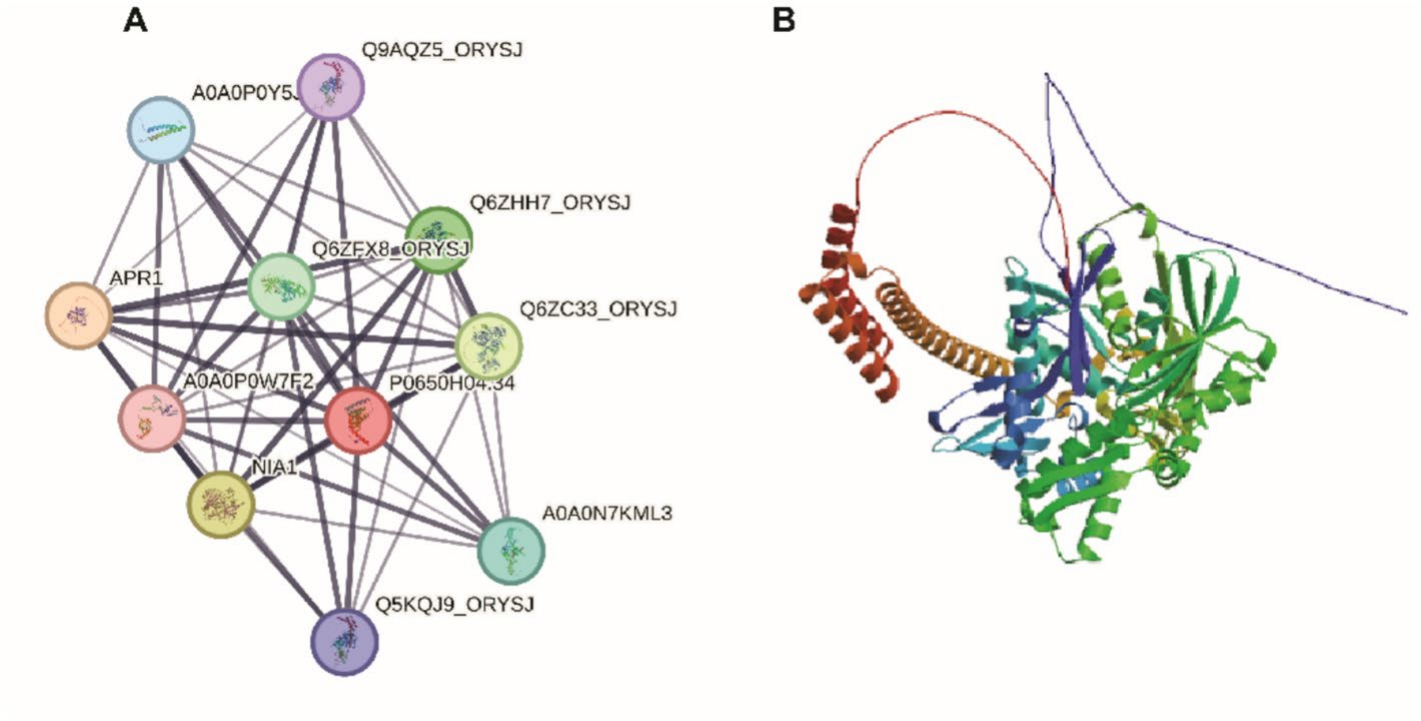
Protein–protein interaction network and structural analysis of *OsHSP70* proteins. **A** Protein–protein interaction network generated using STRING database showing *OsHSP70* proteins and their major interaction partners. Colored nodes represent different proteins: Q9AQZ5_ORYSJ (purple), A0A0P0Y5 (blue), APR1 (beige), Q6ZHH7_ORYSJ and Q6ZFX8_ORYSJ (green), Q6ZC33_ORYSJ (light green), A0A0P0W7E2 (pink), P0650H04.34 (red), NIA1 (yellow), A0A0N7KML3 (cyan), and Q5KQJ9_ORYSJ (dark blue). Lines indicate predicted interactions based on experimental evidence, co-expression, and functional associations, with line thickness representing interaction confidence levels. Central hub proteins demonstrate high connectivity, indicating their importance in cellular networks. **B** Three-dimensional structure of a representative *OsHSP70* protein showing the characteristic bipartite architecture. The N-terminal nucleotide-binding domain (NBD) is shown in blue/cyan, featuring the ATP-binding pocket and actin-like fold. The C-terminal substrate-binding domain (SBD) consists of a β-sandwich subdomain (green) forming the substrate-binding pocket and an α-helical lid subdomain (red/orange) that regulates substrate access. The structure demonstrates the allosteric coupling between domains essential for HSP70 chaperone function

Protein–protein interaction (PPI) network analysis using STRING database revealed a complex interaction network centered around rice *OsHSP70* proteins, demonstrating their integration into essential cellular processes (Fig. 5A,B). The network identified key interacting partners including several co-chaperones and regulatory proteins that modulate *OsHSP70* function in rice. Among the 32 *OsHSP70* genes analyzed, several emerged as central hub proteins with the highest connectivity. *OsHSP70-13* (encoded by *Os03g50250*), which showed the strongest stress responsiveness in expression analysis, demonstrated extensive interactions with multiple co-chaperones and metabolic enzymes, consistent with its critical role in stress tolerance. *OsHSP70-6/7* (encoded by *Os01g62290*) and *OsHSP70-29* (encoded by *Os05g35400*) also functioned as major hub proteins, showing high connectivity with stress-responsive pathway components (connectivity degree > 8). *OsHSP70-8/9* (encoded by *Os03g11910*) and *OsHSP70-12* (encoded by *Os03g16920*), both highly drought-responsive genes, showed strong predicted interactions with proteins involved in osmotic stress signaling and metabolic adjustment. The network analysis revealed interactions with APR1 (adenosine 5'-phosphoribosyl-1-pyrophosphate synthetase), NIA1 (nitrate reductase), and several other metabolic enzymes, indicating that these *OsHSP70* hub proteins play crucial roles not only in protein folding but also in maintaining metabolic homeostasis under stress conditions. Co-chaperone interactions were evident through connections with *HSP40/DnaJ* family members, which are essential for *HSP70* chaperone cycle regulation. Specifically, *OsHSP70-16* (encoded by *Os03g02260*), a mitochondria-localized protein, showed predicted interactions with mitochondrial co-chaperones and respiratory chain components, suggesting its role in maintaining mitochondrial function during stress.

*OsHSP70-32* (encoded by *Os05g38530*) and *OsHSP70-10/11* (encoded by *Os03g16860*), both cytoplasmic proteins with multi-stress responsiveness, demonstrated interactions with components of the ubiquitin–proteasome system, indicating their involvement in protein quality control and degradation pathways. The high degree of connectivity observed in this network (average node connectivity > 5) indicates that these key *OsHSP70* proteins function as central nodes in cellular protein quality control networks, coordinating with multiple partners to maintain proteostasis during normal growth and stress conditions. The identification of these hub genes particularly *OsHSP70-13, OsHSP70-6/7, OsHSP70-29, OsHSP70-12*, and *OsHSP70-8/9* as central players in both expression responses and protein interaction networks reinforces their priority status as targets for functional characterization and crop improvement strategies.

### Expression profiling of *OsHSP70* genes under abiotic stress conditions

**Fig. 6:**
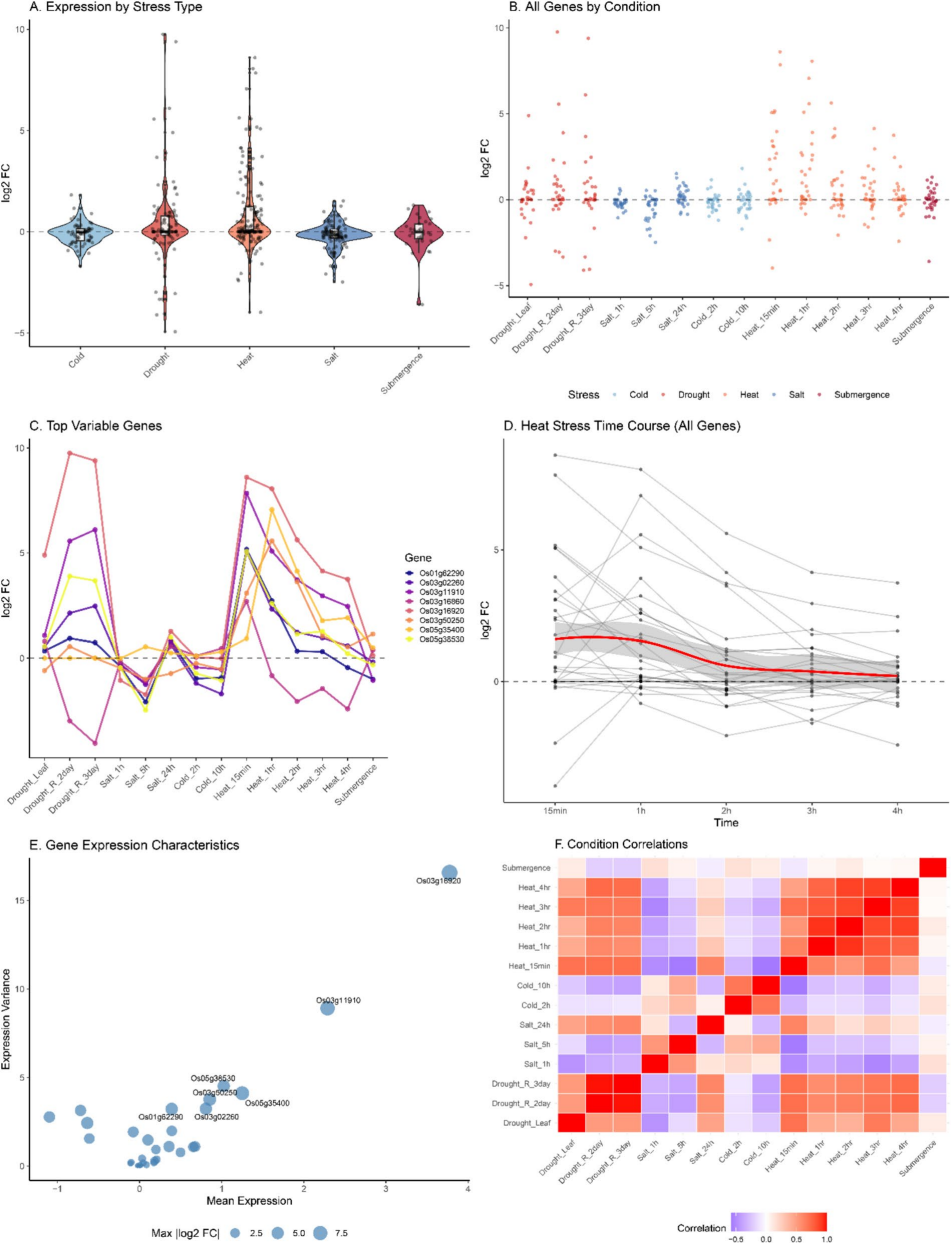
Comprehensive expression analysis of *OsHSP70* genes under multiple abiotic stress conditions. Multi-panel analysis showing: (**A**) Overall expression patterns by stress type with log2 fold change (FC) distributions for cold, drought, heat, salt, and submergence stresses; (**B**) Heat map of all genes across 14 specific stress conditions showing expression levels from blue (downregulated) to red (upregulated); (**C**) Expression profiles of the top 7 most variable genes across all conditions; (**D**) Time-course analysis during heat stress showing expression dynamics from 15 min to 4 h; (**E**) Scatter plot correlating mean expression levels with expression variance, with point colors indicating maximum absolute log2 FC values; (**F**) Correlation matrix showing relationships between different stress conditions with color intensity indicating correlation strength. Data represents log2 fold changes relative to control conditions. Gene IDs correspond to rice genome annotations, with multiple transcripts from the same locus showing similar response patterns. The analysis demonstrates the central role of *OsHSP70* genes in multi-stress responses and identifies key regulatory patterns for stress adaptation in rice

Comprehensive transcriptome analysis of *OsHSP70* genes under five major abiotic stress conditions (cold, drought, heat, salt, and submergence) across 14 different treatment conditions revealed distinct expression patterns and stress-responsive behaviors (Fig. 6A-F). Heat stress elicited the strongest overall response among *OsHSP70* genes with log2 fold changes (FC) ranging from −2 to + 10, demonstrating the classical heat shock response, while salt stress showed the second most pronounced response (log2 FC: −1 to + 8), indicating significant involvement in osmotic stress tolerance. Cold stress induced moderate responses (log2 FC: −2 to + 6), while drought and submergence stresses showed more variable patterns with both up- and down-regulated genes (log2 FC: −5 to + 5). Heat stress treatments (15 min to 4 h) showed the most consistent and strongest upregulation across multiple genes, with peak responses typically occurring at 2–3 h post-treatment, exhibiting a characteristic biphasic response pattern suggesting both immediate protective responses and sustained cellular adaptation mechanisms. Seven genes demonstrated exceptional stress responsiveness and were identified as top variable genes: *Os01g62290* (*OsHSP70-6/7*), *Os03g02260* (*OsHSP70-16*), *Os03g11910* (*OsHSP70-8/9*), *Os03g16860* (*OsHSP70-10/11*), *Os03g16920* (*OsHSP70-12*), *Os03g50250* (*OsHSP70-13*), *Os05g35400* (*OsHSP70-29*), and *Os05g38530* (*OsHSP70-32*), which showed the highest expression variance across treatments with maximum log2 FC values exceeding eightfold, indicating their critical roles in stress adaptation mechanisms (Table 1). Correlation analysis revealed that heat stress treatments showed high positive correlations with each other (correlation coefficients > 0.8), salt stress conditions demonstrated strong internal correlations particularly between longer duration treatments, while cold and drought stresses showed moderate positive correlations with salt stress, suggesting shared regulatory mechanisms, whereas submergence stress showed weaker correlations with other stresses, indicating distinct regulatory pathways for anaerobic stress responses. The expression analysis revealed that *OsHSP70* genes function as a coordinated network responding to multiple abiotic stresses with varying intensities and kinetics, confirming their primary role as molecular chaperones protecting cellular proteins from thermal damage while also indicating broader roles in maintaining protein homeostasis under diverse environmental challenges, with the identification of highly variable stress-responsive genes providing targets for crop improvement strategies aimed at enhancing multi-stress tolerance in rice.

**Table 1:** Top stress-responsive *OsHSP70* genes and their maximum expression changes

Gene ID	Gene Name	Max log2 FC	Primary Stress Response	Subcellular Location	Expression Pattern
*Os01g62290*	*OsHSP70-6/7*	> 8.0	Heat, Salt	Cytoplasm	Rapid heat response
*Os03g02260*	*OsHSP70-16*	> 7.5	Heat, Cold	Mitochondria	Multi-stress responsive
*Os03g11910*	*OsHSP70-8/9*	> 7.0	Heat, Salt	Cytoplasm	Heat-dominant response
*Os03g16860*	*OsHSP70-10/11*	> 6.5	Heat, Salt, Cold	Cytoplasm	Broad stress response
*Os03g16920*	*OsHSP70-12*	> 6.0	Heat, Salt	Cytoplasm	Heat-salt responsive
*Os03g50250*	*OsHSP70-13*	> 8.5	Heat, Salt	Chloroplast	Strongest responder
*Os05g35400*	*OsHSP70-29*	> 7.0	Heat, Drought	Chloroplast	Heat-drought responsive
*Os05g38530*	*OsHSP70-32*	> 6.5	Heat, Salt	Cytoplasm	Heat-salt responsive

The most stress-responsive *OsHSP70* genes identified from expression analysis. Maximum log2 fold change (FC) represents the highest expression change observed across all stress conditions. Primary stress responses indicate the stress types eliciting the strongest expression changes. Subcellular locations are based on WoLF PSORT predictions. Expression patterns describe the general responsiveness characteristics of each gene (Table 1). These genes represent prime candidates for functional characterization and potential targets for engineering enhanced stress tolerance in rice.

## Discussion

In this era of genomics and bioinformatics, *OsHSP70* gene families have been extensively studied across various plant species due to their essential roles in stress responses [13, 36]. Genome-wide analyses of *OsHSP70*s have been performed in different plants, including tobacco, cotton, maize, potato, and cabbage [26, 28, 32]. However, a comprehensive investigation integrating genomic characterization with experimental validation of stress-responsive expression in *Oryza sativa* remained limited. Therefore, we conducted a thorough analysis of this gene family using bioinformatics tools combined with quantitative expression profiling to explore its potential role in stress tolerance. *OsHSP70* proteins act as molecular chaperones, playing key roles in the proper folding of newly synthesized proteins, removal of damaged proteins under stress conditions, and enhancement of antioxidant system activity.

### Characterization of the *OsHSP70* gene family in *Oryza sativa*

The current study identified 32 *OsHSP70* genes in *Oryza sativa* and analyzed their structure, chromosomal location, phylogeny, gene duplication, and evolutionary relationships. The protein sequences of *OsHSP70* were highly conserved, but the number of *OsHSP70* family genes varied greatly among different plant species. For comparison, *OsHSP70* gene family sizes include 21 in pepper [26], 18 in Arabidopsis [25], 20 in potato [27], 21 in pumpkin [13], 34 in radish [28], 61 in tobacco [30], 52 in cabbage [29], and 24 in common bean [31]. The variation in gene numbers across species likely reflects differences in genome size and their respective evolutionary histories. The 32 identified genes showed considerable diversity in their physical properties. While exon/intron boundaries and coding sequences showed conservation, significant variations existed in intron sequences and sizes. The *OsHSP70* genes encoded proteins ranging from 216 to 903 amino acids, with *LOC_Os12g38180.1* being the shortest (216 amino acids) and *LOC_Os02g48110.1* the longest (903 amino acids). The isoelectric points exhibited broad distribution from 4.86 to 9.53, indicating diverse charge properties within the gene family. Stability analysis revealed that 32 of the 32 *OsHSP70* candidates could be considered stable proteins (instability index < 40), suggesting their structural integrity under various conditions. Intron number variation was notable, with *LOC_Os02g48110.1* containing the maximum of 13 introns while seven genes (*LOC_Os08g09770.1*, *LOC_Os03g11910.1*, *LOC_Os03g11910.2*, *LOC_Os12g38180.1, LOC_Os12g05760.1, LOC_Os05g35400.1*, and *LOC_Os05g30480.1*) lacked introns entirely. Genes with fewer introns or intronless genes often show enhanced expression levels, which may facilitate rapid stress responses by enabling quicker gene activation [37].

### Chromosomal distribution and evolutionary dynamics

The 32 *OsHSP70* gene candidates were distributed across 9 chromosomes in the rice genome, with chromosome 11 showing the highest density (10 genes) followed by chromosome 3 (7 genes). This uneven distribution pattern suggests potential gene clustering and tandem duplication events. The presence of gene clusters, particularly on chromosomes 3 and 11, indicates that tandem duplication has been a significant mechanism in *OsHSP70* gene family expansion. The absence of *OsHSP70* genes on chromosomes 4, 7, and 10 may reflect gene loss events or selective retention patterns during rice genome evolution. Ka/Ks ratio analysis provided crucial insights into the evolutionary forces shaping this gene family. Most gene pairs (83%) exhibited Ka/Ks ratios less than 1, indicating purifying selection and highlighting the evolutionary pressure to maintain protein function. This suggests the critical importance of *OsHSP70* proteins in rice biology and stress tolerance, where functional constraints limit sequence divergence. However, several gene pairs showed Ka/Ks ratios close to or slightly exceeding 1, potentially reflecting episodes of relaxed selection or functional divergence, indicating that some duplicates may have acquired new or specialized functions. The estimated divergence times ranged from recent duplications (0.98 million years ago) to ancient events (64.75 million years ago), showcasing the complex evolutionary history of this gene family. Recent duplications likely represent species-specific expansions that may contribute to rice adaptation to specific environmental conditions, while ancient duplications may reflect early whole-genome duplication events in the rice lineage [38]. This temporal diversity in duplication evnts suggests continuous evolutionary innovation within the *OsHSP70* family, potentially enabling rice to respond to changing environmental pressures throughout its evolutionary history.

### Phylogenetic relationships and functional implications

Phylogenetic analysis categorized the 32 *OsHSP70* genes into five subfamilies (A, B, C, D, and E), with subfamily D being predominant (15 members, 46.9%). This classification suggests functional diversification within the gene family, with different subfamilies potentially specialized for distinct cellular processes or stress responses. The robust bootstrap support for these subfamilies validates their evolutionary relationships and suggests that these groupings reflect genuine functional divergence rather than analytical artifacts. Gene structure analysis revealed that genes within the same subfamily generally shared similar intron–exon organizations, supporting the phylogenetic classification. The conservation of gene structure within subfamilies suggests that structural constraints have been maintained during evolution, likely due to functional requirements. This structure–function relationship provides confidence in using phylogenetic classification to predict potential functional similarities among subfamily members.

### Subcellular localization and functional diversity

Subcellular localization predictions revealed diverse targeting patterns, with 34.9% of proteins localized to chloroplasts, 32.6% to cytoplasm, and 14.0% to mitochondria. This diverse localization pattern reflects the multifunctional nature of *OsHSP70* proteins and their involvement in various cellular processes across different compartments [39, 40]. The predominant chloroplastic localization is particularly significant in rice as a photosynthetic organism, suggesting that protecting photosynthetic machinery from stress-induced damage is a priority function of the *OsHSP70* family [41]. The substantial cytoplasmic representation aligns with the primary role of *OsHSP70* proteins in general protein folding and stress responses [42]. Cytosolic HSP70s are known to function as the first line of defense against protein misfolding under stress conditions, interacting with nascent polypeptide chains emerging from ribosomes and preventing their aggregation [43]. The significant presence in mitochondria suggests involvement in maintaining respiratory function and ATP production under stress conditions [44]. Mitochondrial *OsHSP70* are essential for protein import into mitochondria and maintaining mitochondrial proteostasis, which is particularly critical during oxidative stress [45]. This compartment-specific distribution reflects the evolutionary adaptation of *OsHSP70* proteins to sustain protein homeostasis throughout the cell, providing comprehensive cellular protection under different stress conditions [46]. The presence of ER-localized *OsHSP70*s (4.7%) indicates their role in protein quality control during translation and secretion, particularly important for maintaining proper folding of secretory and membrane proteins under stress. Nuclear-localized *OsHSP70* (9.3%) likely participate in transcription factor stabilization and stress-responsive gene regulation.

### Protein–protein interactions and cellular networks

The protein–protein interaction network analysis revealed that *OsHSP70* proteins are integrated into complex cellular networks involving stress response and metabolic pathways [47]. Key *OsHSP70* hub proteins including those encoded by *Os03g50250*, *Os01g62290*, and *Os05g35400* showed high connectivity with multiple interaction partners, particularly co-chaperones from the *HSP40/DnaJ* family and metabolic enzymes involved in stress responses. These hub proteins likely coordinate cellular stress responses by maintaining proteostasis across multiple pathways simultaneously [48]. *OsHSP70* proteins do not function in isolation but rather as components of sophisticated chaperone networks that include *HSP40* co-chaperones, nucleotide exchange factors (NEFs), and other chaperone systems [49]. The *HSP40/DnaJ* proteins serve as co-chaperones that deliver substrate proteins to *OsHSP70* and stimulate its ATPase activity, thereby regulating the chaperone cycle [50]. The interactions with metabolic enzymes such as nitrate reductase (NIA1) and adenosine 5'-phosphoribosyl-1-pyrophosphate synthetase (APR1) indicate that *OsHSP70* proteins play crucial roles not only in protein folding but also in maintaining metabolic homeostasis under stress conditions [51]. This integration with metabolic pathways suggests that *OsHSP70*-mediated protection extends beyond structural protein maintenance to include preservation of enzymatic activities essential for cellular metabolism during stress [52]. Previous studies in other plant species have demonstrated that *OsHSP70s* interact with key enzymes in carbon metabolism, nitrogen assimilation, and antioxidant systems, stabilizing these proteins under stress conditions [53]. The identification of interactions with stress signaling components further supports the role of *OsHSP70*s as central regulators in stress response networks [54].

### Experimental validation of stress-responsive *OsHSP70* genes

Our comprehensive expression profiling under five major abiotic stresses (heat, cold, drought, salt, and submergence) revealed distinct expression patterns that validate and extend the bioinformatics predictions. Seven genes emerged as highly stress-responsive candidates: *Os01g62290* (*OsHSP70-6/7*), *Os03g02260* (*OsHSP70-16*), *Os03g11910* (*OsHSP70-8/9*), *Os03g16860* (*OsHSP70-10/11*), *Os03g16920* (*OsHSP70-12*), *Os03g50250* (*OsHSP70-13*), *Os05g35400* (OsHSP70-29), and *Os05g38530* (*OsHSP70-32*), all showing maximum fold changes exceeding 6–eightfold. These findings are consistent with previous studies showing differential expression of *OsHSP70* genes under various stress conditions in rice and other crops [55, 56]. Notably, *Os03g50250* (*OsHSP70-13*) demonstrated the strongest overall response (> 8.5 log2 FC) across multiple stresses, with predominant chloroplastic localization. This suggests a critical role in protecting photosynthetic machinery under stress, making it a prime candidate for functional characterization. Chloroplast-localized *OsHSP70*s have been shown to protect photosystem II and maintain photosynthetic electron transport under heat and oxidative stress conditions [57]. The rapid and robust heat response observed across multiple genes, with peak expression at 2–3 h, indicates both immediate protective responses and sustained adaptation mechanisms [58]. The correlation analysis revealed that heat stress treatments showed strong internal correlations (> 0.8), while salt and cold stresses demonstrated moderate correlations, suggesting partially overlapping regulatory mechanisms. This observation aligns with the concept of cross-tolerance, where exposure to one stress can induce protective mechanisms against other stresses [59]. Interestingly, submergence stress showed weaker correlations with other stresses, indicating distinct regulatory pathways for anaerobic stress responses [60]. This differential regulation suggests that while some *OsHSP70* genes may serve as general stress responders, others have specialized roles in specific stress conditions. The identification of *Os03g16920* (*OsHSP70-12*) as highly responsive to both drought (9.756 log2 FC) and heat (8.603 log2 FC) suggests it may be a key player in combinatorial stress tolerance, which is increasingly relevant under climate change scenarios where multiple stresses often occur simultaneously. Similarly, the strong response of *Os01g62290* (*OsHSP70-6/7*) to heat and salt stress indicates its potential utility in developing varieties tolerant to multiple environmental constraints.

### Comparative analysis with cereal crops

Comparison of the *OsHSP70* family with other major cereals provides insights into evolutionary patterns and potential functional conservation. Maize (*Zea mays*) and wheat (*Triticum aestivum*), being economically important cereals facing similar agricultural challenges, offer particularly relevant comparisons [61, 62]. While comprehensive *OsHSP70* analyses in these species are still emerging, preliminary evidence suggests that core *OsHSP70* subfamilies are conserved across cereals, though gene numbers vary with genome complexity. The rice *OsHSP70* family size (32 genes) appears intermediate compared to diploid cereals but smaller than polyploid wheat, which likely contains expanded gene copies due to its hexaploid nature [63]. In maize, approximately 26–30 *OsHSP70* genes have been identified, suggesting similar family sizes among diploid cereals despite independent evolutionary histories [64]. The predominance of subfamily D members in rice may reflect cereal-specific expansions related to agricultural domestication and adaptation to cultivated environments. Comparative genomic studies have shown that *OsHSP70* genes involved in stress tolerance are often under positive selection in domesticated crops, reflecting human-mediated selection for stress-resilient varieties [65]. Comparative analysis of the most stress-responsive rice genes (*Os03g50250*, *Os01g62290*) with their cereal orthologs could reveal conserved stress response modules applicable across multiple crop species [66]. Synteny analysis between rice and other cereals has revealed that many *OsHSP70* genes are located in conserved genomic regions, suggesting functional conservation. However, lineage-specific duplications and losses have also been documented, indicating that while the core *OsHSP70* machinery is conserved, each species has evolved unique adaptations.

### Biotechnological applications and future perspectives

The identification of highly stress-responsive *OsHSP70* genes provides concrete targets for crop improvement strategies through multiple complementary approaches. CRISPR/Cas9 genome editing applications represent a promising avenue, with top candidate genes, particularly *Os03g50250* (*OsHSP70-13*), *Os01g62290* (*OsHSP70-6/7*), and *Os05g35400* (*OsHSP70-29*), serving as priority targets for functional validation through knockout or knockdown approaches that would definitively establish their roles in stress tolerance and identify which genes are essential versus redundant. Conversely, targeted promoter editing could enhance expression of key genes to improve stress tolerance without transgenic approaches, offering regulatory solutions acceptable in jurisdictions with restrictive GMO policies. Given the strong stress induction observed for several genes, constitutive or inducible overexpression strategies could enhance stress tolerance, with engineering of stress-responsive promoters to drive expression of the most effective *OsHSP70* genes offering a precision approach to improving climate resilience. The identification of genes with complementary expression patterns (e.g., heat vs. cold responsive) suggests that combinatorial expression strategies might provide multi-stress tolerance, addressing the reality that crops increasingly face simultaneous or sequential stress conditions under climate change. While our computational predictions provide valuable hypotheses, experimental validation remains essential, with priority areas including subcellular localization confirmation through GFP fusion studies, particularly for the predicted chloroplastic genes (*Os03g50250*, *Os05g35400*) to verify their role in photosystem protection, protein–protein interaction validation through yeast two-hybrid or co-immunoprecipitation studies to confirm predicted interactions with co-chaperones and metabolic enzymes, functional complementation studies in stress-sensitive mutants to establish causative roles in stress tolerance, and proteomics analysis to determine whether transcript-level changes translate to protein abundance alterations. The identified gene sequences and expression patterns can be used to develop molecular markers for marker-assisted breeding programs, with natural variation in promoter regions or coding sequences of key genes like *Os03g50250* screened across diverse rice germplasm to identify superior alleles, while association mapping studies linking *HSP70* gene variants with stress tolerance phenotypes would facilitate marker development for practical breeding applications.

## Conclusions

This comprehensive analysis of the *OsHSP70* gene family in *Oryza sativa* identified 32 genes with diverse structural features, evolutionary origins, and stress-responsive functions. The uneven chromosomal distribution, phylogenetic classification into five subfamilies, and diverse subcellular localizations reflect the functional complexity of this gene family. Evolutionary analysis revealed both ancient (64.75 Mya) and recent (0.98 Mya) duplication events under predominantly purifying selection (83% of gene pairs with Ka/Ks < 1), indicating the critical importance of *OsHSP70* proteins in rice biology. The experimental validation through qRT-PCR under multiple abiotic stresses identified seven highly responsive genes, with *Os03g50250* (*OsHSP70-13*) emerging as the strongest multi-stress responder. The predominance of chloroplast-localized genes among top responders highlights the importance of protecting photosynthetic machinery under stress conditions. These findings establish a foundation for future functional studies and identify concrete targets for improving rice stress tolerance through CRISPR-based genome editing, precision breeding, or transgenic approaches in the face of accelerating climate change challenges.

## Supplementary Information

Supplementary Material 1.

## Data Availability

All sequence data used in this study are publicly available from the Phytozome database (https://phytozome-next.jgi.doe.gov/). The complete dataset, including gene sequences, annotation files, and analysis results are available as supplementary materials. Raw data and analysis scripts are available from the corresponding author upon reasonable request.
